# Complete mitochondrial genome of Eurasian Hobby *Falco subbuteo* (Falconiformes:Falconidae) and phylogenetic analyses

**DOI:** 10.1080/23802359.2021.1907247

**Published:** 2021-03-26

**Authors:** Xue Gou, Shize Li, Cheng Wang, Caichun Peng, Canshi Hu, Mingming Zhang, Haijun Su

**Affiliations:** a Forestry College, Guizhou University, Guiyang, China; b Research Center for Biodiversity and Natural Conservation, Guizhou University, Guiyang, China; c College of Life Sciences, Guizhou University, Guiyang, China; d Department of Food Science and Engineering, Moutai Institute, Renhuai, China

**Keywords:** Complete genome, gene arrangement, Eurasian Hobby, mitochondrial DNA, *Falco subbuteo*

## Abstract

The Eurasian Hobby *Falco subbuteo* is a *Falco* bird belonging to the group Falconiformes, and was listed in Appendices II of the Convention on International Trade in Endangered Species of Wild Fauna and Flora and listed as a Class II protected species on China's List of Wildlife under Special State Protection. In this study, the complete mitogenome of *F. subbuteo* was determined. The mitochondrial DNA is packaged in a compact 17,678 based pair (bp) circular molecule with A + T content of 54.70%. It contains 37 typical mitochondrial genes, including 13 protein-coding genes, 2 rRNAs and 22 tRNAs, and 2 non-coding regions (D-Loop). We reconstructed a phylogenetic tree based on mitogenome sequences of 13 Falconidae species and one outgroup. Phylogenetic analysis indicated that *F. subbuteo* was a sister taxon to *F. cuvierii* with node support 100. The new mitogenome data would provide useful information for application in conservation genetics and further clarify the phylogenetic evolution of this species.

The Eurasian Hobby *Falco subbuteo* is a *Falco* bird belonging to the group Falconiformes, and was listed in Appendices II of the Convention on International Trade in Endangered Species of Wild Fauna and Flora and listed as a Class II protected species on China's List of Wildlife under Special State Protection. which breeds in Eurasia, Northwest Africa and overwinters in Japan, India, Laos, Myanmar. Especially, it distributes almost all over the country in China. They are living in different types of forests, shrubbery's, forest edges, water areas or near inhabitants. They live solitarily or in pairs, fly very rapidly and prey on small mammals such as rodents, young birds and also insects. Their food habit is very beneficial to the preservation of forests and the regulation of the relative equilibrium of forest ecosystems (Xu 1995; Wang and Chen 1998). Up to now, no complete mitochondrial genome data of *F. subbuteo* is available in the GenBank. In this study, we sequenced the complete mitochondrial genome of *F. subbuteo* (GenBank number: MW266990) and examined its phylogenetic relationship with other Falconiformes species whose mtDNA data is available.

The tissues were collected from an accidental death of individual on 15 November 2019, found in Shiqian country, Guizhou Province, China (E108°12′44.74″, N27°30′15.55″). It was kept in the research Center for Biodiversity and Natural Conservation, Guizhou University. The stored number of the sample is GZUNZ20201118001.The extraction was performed using DNA Rapid Extraction Kit (Beijing Aidlab Biotechnologies Co., Ltd) according to the kit manual. The mitochondrial genomes of *Falco tinnunculus* (EU196361.1) was used to design primers for polymerase chain reaction (PCR) and used as template for gene annotation.

The complete mtDNA sequences of the Eurasian Hobby was 17,678 bp in length, It was overall base composition is: A, 32.33%; C, 31.92%; G, 13.38% and T, 22.37%. The A + T content was 54.70%, which is within the range for avian mitogenomes (51.6–55.7%; Haring et al. 2001). It has a typical circular mitochondrial genome containing 13 protein-coding genes, 22 transfer RNAs, 2 ribosomal RNAs and 2 non-coding A + T-rich region, which are usually found in birds (Boore 1999). The gene order was consistent with the standard avian gene order (Gibb et al. 2007). Of the 13 protein-coding genes, 11 utilize the standard mitochondrial start codon ATG, however COI use GTG, ND3 uses ATA as the initiation codon. TAA is the most frequent stop codon, although COI end with AGG, ND5 end with AGA, ND6 end with TAG, ND4 and COIII stop with the single nucleotide T (Table 1). The 12S rRNA is 981 bp, and the 16S rRNA is 1597 bp in length, which are located between tRNA-Phe and tRNA-Leu, and separated by tRNA-Val. All tRNAs possess the classic clover leaf secondary structure, as observed in other bird mitogenomes (Bernt et al. 2013). Most of the mitochondrial genes are encoded on heavy strand (H-strand) except for ND6 and eight tRNA genes, which are encoded on light strand (L-strand) (Table 1).

**Table 1.** Organization of the complete mitochondrial genome of Eurasian Hobby *Falco subbuteo*.

	Position			Codon			
Gene	Start-End	Size	Spacer (+) or Overlap (–)	Start	Stop	Anti-codon	Strand
tRNA-Phe	1–73	73				GAA	H
12S rRNA	74–1054	981					H
tRNA-Val	1055–1127	73				TAC	H
16S rRNA	1128–2724	1597					H
tRNA-Leu	2725–2798	74				TAA	H
ND1	2814–3788	975	15	ATG	TAA		H
tRNA-Ile	3804–3876	73	15			GAT	H
tRNA-Gln	3886–3956	71	9			TTG	L
tRNA-Met	3956–4027	72	–1			CAT	H
ND2	4029–5069	1041	1	ATG	TAA		H
tRNA-Trp	5069–5146	78	–1			TCA	H
tRNA-Ala	5157–5226	70	10			TGC	L
tRNA-Asn	5237–5309	73	10			GTT	L
tRNA-Cys	5312–5378	67	2			GCA	L
tRNA-Tyr	5378–5448	71	–1			GTA	L
COI	5450–7000	1551	1	GTG	AGG		H
tRNA-Ser	6992–7065	74	–9			TGA	L
tRNA-Asp	7068–7136	69	2			GTC	H
COII	7145–7828	684	8	ATG	TAA		H
tRNA-Lys	7830–7899	70	1			TTT	H
ATP8	7901–8068	168	1	ATG	TAA		H
ATP6	8059–8742	684	–10	ATG	TAA		H
COIII	8742–9525	784	–1	ATG	T		H
tRNA-Gly	9526–9594	69				TCC	H
ND3	9595–9946	352	1	ATA	TAA		H
tRNA-Arg	9948–10,016	69	1			TCG	H
ND4L	10,018–10,314	297	1	ATG	TAA		H
ND4	10,308–11,685	1378	–7	ATG	T		H
tRNA-His	11,686–11,756	71				GTG	H
tRNA-Ser	11,757–11,822	66				GCT	H
tRNA-Leu	11,822–11,881	60	–1			TAG	H
ND5	11,893–13,710	1818	11	ATG	AGA		H
Cytb	13,717–14,859	1143	6	ATG	TAA		H
tRNA-Thr	14,862–14,930	69	2			TGT	H
D-LOOP	14,931–16,365	1435					
tRNA-Pro	16,366–16,434	69	1435			TGG	L
ND6	16,450–16,971	522	15	ATG	TAG		L
tRNA-Glu	16,975–17,045	71	3			TTC	L
D-LOOP	17,046–17,678	633					

We used PhyML v. 3.0 to construct the phylogenetic tree using the maximum-likelihood method (Figure 1) based on the mitogenome sequences of *F. subbuteo* of other 13 Falconidae species and one *Microhierax melanoleucos* as outgroup. The result showed that *F. subbuteo* was a sister taxon to *F. cuvierii* with node support 100 (Figure 1).

**Figure 1.**
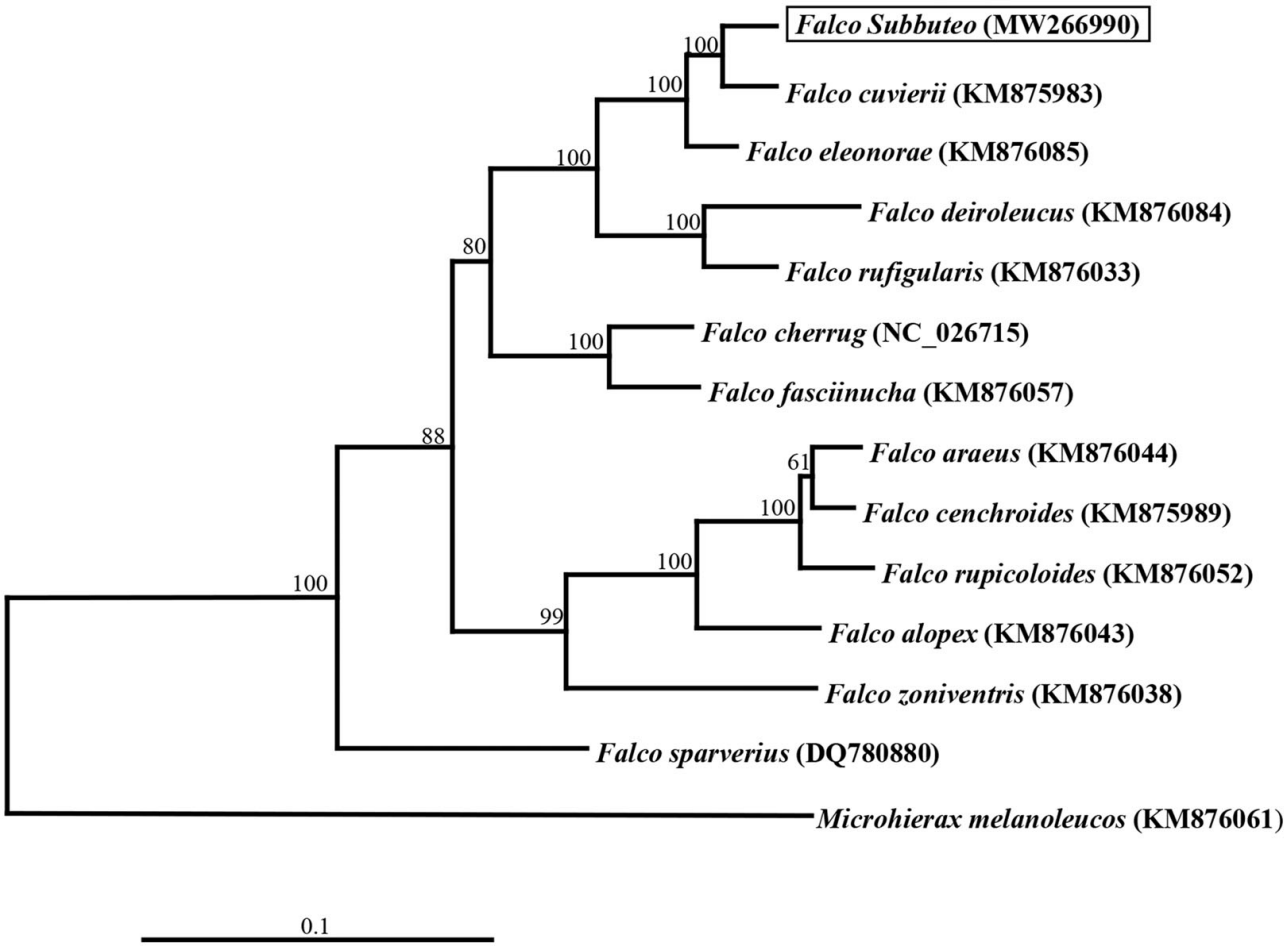
Maximum likelihood tree based on mitogenome sequences of 13 Falconidae species and one outgroup. Numbers nodes are bootstrap supports.

This study is the first to report and analyze the complete mitochondrial genome of *F. subbuteo*. The complete mitogenome of *F. subbuteo* is in favor of conservation of the nationally protected species, and provides fundamental genetic data for the evolutionary research of Falconidae.

## Data Availability

The genome sequence data that support the findings of this study are openly available in GenBank of NCBI at (https://www.ncbi.nlm.nih.gov/) under the accession no. MW266990. The associated Figure (https://doi.org/10.6084/m9.figshare.13377665).
